# Elucidating the mechanism of cefpodoxime-BSA interaction via a combination of multi-spectroscopic methods and molecular docking simulations

**DOI:** 10.1038/s41598-026-39137-8

**Published:** 2026-03-01

**Authors:** Reem N. El Gammal, Heba Elmansi, Ali A. El-Emam, Mohammed E. A. Hammouda, Fathalla Belal

**Affiliations:** 1 Department of Medicinal Chemistry, Faculty of Pharmacy, Mansoura University, 35516 Mansoura, Egypt; 2 Pharmaceutical Analytical Chemistry Department, Faculty of Pharmacy, Mansoura University, Mansoura, 35516 Egypt; 3 Department of Pharmaceutical Chemistry, Faculty of Pharmacy, Horus University - Egypt (HUE), New Damietta, Egypt

**Keywords:** Cefpodoxime, Bovine serum albumin, Multi-spectroscopic, Biochemistry, Biophysics, Chemistry

## Abstract

This research provides a comprehensive elucidation of the molecular interaction between cefpodoxime (CFP) and bovine serum albumin (BSA) through an integrated approach combining computational docking and a suite of spectroscopic methods. Initial evidence from UV-Vis absorption spectroscopy confirmed a ground-state complex formation between the drug and the protein. Subsequent fluorescence quenching studies established a static quenching mechanism, with a binding constant (Kb) of 3.99 × 10^4^ L·mol^−1^ determined at 298 K, indicating moderate binding affinity. Analysis of the thermodynamic parameters, computed via the Van’t Hoff equation, revealed that the binding process is both spontaneous and endothermic. The positive entropy change (ΔS°) identified hydrophobic interactions as the predominant driving force for the complex formation.

The binding site was precisely localized to subdomain IIA (Site I) of BSA, a finding consistently supported by two independent lines of evidence: competitive site-marker displacement assays and molecular docking simulations. Collectively, these insights into the binding affinity, forces, and specific location are fundamental for advancing the understanding of CFP’s pharmacokinetic profile. This knowledge is critical for predicting its distribution and elimination in vivo, thereby informing its safe clinical use and helping to mitigate potential adverse effects.

**Supplementary Information:**

The online version contains supplementary material available at 10.1038/s41598-026-39137-8.

## Introduction

The transport of small molecules in the bloodstream is mediated by serum albumin, the circulatory system’s predominant protein, which forms specific molecular interactions^1^. In-vitro research frequently utilizes Bovine Serum Albumin (BSA) as a model, given its well-documented structural comparability to Human Serum Albumin (HSA). The BSA polypeptide chain (583 amino acids, 66.5 kDa) is organized into three linear domains, each containing two subdomains (A and B). Hydrophobic cavities within subdomains IIA (Site I) and IIIA (Site II) serve as the primary binding pockets for ligands^2–4^. The formation of a stable complex at these sites is a key focus for elucidating fundamental drug-plasma binding principles^5^.

A drug’s pharmacokinetic profile is primarily determined by the interaction it has with serum albumin^6,7^. Serum albumin binding affinity is a key regulator of a drug’s free and bound plasma concentrations. The equilibrium between these states is fundamental to the drug’s mechanism of action, underscoring the necessity of elucidating its precise binding behavior with albumin^8^. Understanding these binding dynamics is therefore not merely an academic exercise but a key item of pharmaceutical development. By systematically investigating a compound’s interaction with serum albumin, scientists can predict and optimize it’s in vivo behavior, leading to the design of safer and more effective therapeutics with desired pharmacokinetic properties.

Cefpodoxime (CFP) is a third-generation cephalosporin antibiotic that is taken orally and has a broad spectrum of action. It is given as cefpodoxime proxetil, a prodrug that, after absorption, hydrolyzes to the active CFP^9^. Cefpodoxime demonstrates potent in-vitro activity against a range of pathogens. While maintaining its effectiveness against Gram-positive bacteria like streptococci, it is especially powerful against Gram-negative bacteria, such as those that produce beta-lactamases and are resistant to many drugs. As a well-tolerated, pioneering oral third-generation cephalosporin, its clinical applications include the treatment of respiratory and urinary tract infections, tonsillitis, pharyngitis, acute otitis media, and skin structure infections^10^. The structure of cefpodoxime features two critical functional groups that define its properties. At the C-3 site, a methoxymethyl group facilitates its good oral absorption. Concurrently, the presence of a (Z)-2-(2-aminothiazol-4-yl)-(methoxyimino) acetamido moiety at C-7 is directly linked to the antibiotic’s potent Gram-negative coverage and notable β-lactamase stability^10^. Cefpodoxime exhibits a half-life of approximately 1.9 to 2.8 h. Its proxetil tablet formulation has an absolute bioavailability of 50%, which is improved when taken with food but diminished by agents that raise gastric pH, such as antacids or H2-receptor antagonists. The drug demonstrates low plasma protein binding (18–23%), indicating a high potential for tissue distribution. It undergoes minimal metabolism in the body. Unabsorbed drug is degraded within the gastrointestinal tract and eliminated fecally. As a compound cleared predominantly by the kidneys, its pharmacokinetics are significantly affected in patients with renal impairment, characterized by a prolonged half-life and reduced plasma and renal clearance^11^.

**Fig. 1:**
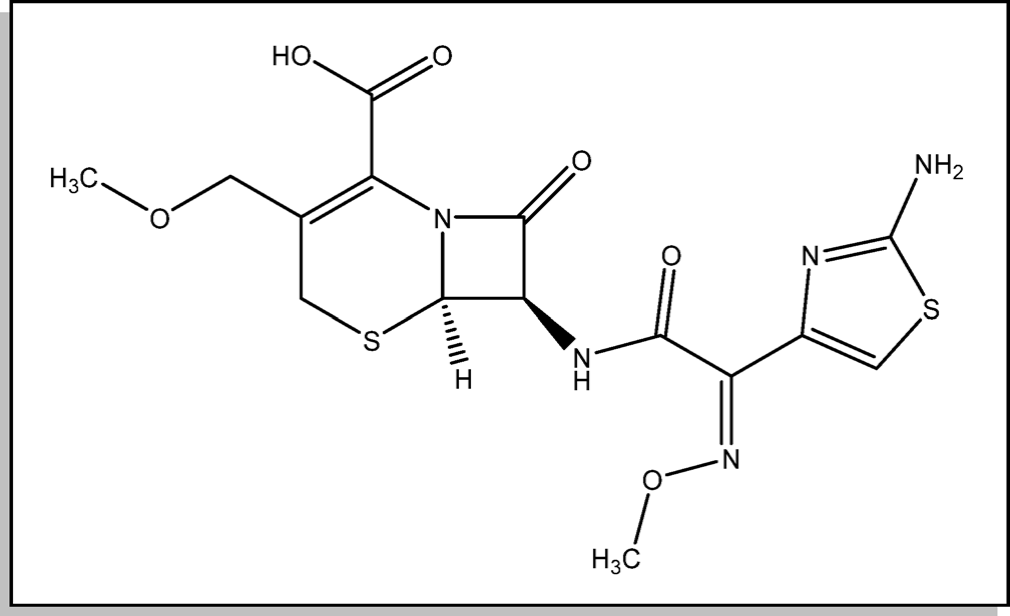
Chemical structure of cefpodoxime (CFP).

To predict pharmacokinetic behavior and reduce potential side effects, a comprehensive understanding of the binding interactions between pharmaceutical compounds and serum proteins is essential. To this end, the present in-vitro research was designed to characterize the binding mechanism of the antibiotic cefpodoxime (CFP) with bovine serum albumin (BSA), a recognized model protein. The investigation employed fluorescence quenching spectroscopy as the principal technique to monitor the interaction (Fig. 1). To obtain a robust thermodynamic profile, the binding constant, the nature of the binding forces, and the number of binding sites were quantified in a physiological buffer (Tris-HCl, pH 7.4) across a temperature range of 298 K to 318 K.

Complementary spectroscopic methods were utilized to probe the structural consequences of this binding. UV-Vis absorption and synchronous fluorescence (SF) spectroscopy provided insights into the complex formation and alterations in the microenvironments of specific aromatic amino acids, respectively. Furthermore, Fourier-transform infrared (FTIR) spectroscopy was used to detect any shifts in the protein’s secondary structure. To corroborate the empirical findings and pinpoint the precise binding location, molecular docking simulations were conducted, providing a three-dimensional model of the CFP-BSA complex.

## Experimental

### Chemicals and reagents

The following materials were sourced for this study: Cefpodoxime (Pharco Pharmaceuticals, Egypt), Diazepam (Amoun Pharmaceutical Co., Egypt), Indomethacin (Medical Union Pharmaceuticals, Egypt), and BSA along with Tris-HCl buffer (Sigma-Aldrich, Germany). The BSA (Lot# SLBM6044V) had a molecular weight of 66.5 kDa.

### Instruments and software

#### Fluorescence measurements

A Cary Eclipse Fluorescence Spectrophotometer (Agilent Technologies, USA) with a Xenon flash lamp was utilized for all fluorimetric analyses. The instrument parameters were set at a detector voltage of 900 V and a smoothing factor of 20. Furthermore, the potential for the inner filter effect (IFE) was studied, as it is an expected phenomenon in fluorescence spectroscopy, and appropriate corrections were applied to the recorded spectra^12^. The fluorescence intensity (FI) was corrected for the inner filter effect (IFE) using the following equation^13^:

$$\:{F}_{cor}={F}_{obs}\:\times\:\:{e}^{({A}_{ex}+{A}_{em})/2}$$

F_cor_: the corrected FI, F_obs_: observed FI,

In this equation, A_ex_ and A_em_ correspond to the total absorbance of CFP at the excitation (285 nm) and emission (λ) wavelengths, respectively.

The observed blue shift in the tryptophan synchronous fluorescence spectrum (Δλ = 60 nm) should be interpreted with caution, as inner filter effects from cefpodoxime absorption in the UV range may influence the spectral shape. While we applied standard fluorescence intensity corrections in quenching experiments, synchronous scans were not explicitly corrected for wavelength-dependent absorption. Nevertheless, the consistency with FTIR and docking results supports the conclusion of a conformational change upon binding.

#### UV-visible spectroscopic measurements

A Shimadzu UV-1601 PC double-beam spectrophotometer (Kyoto, Japan) was used to determine UV-Vis spectrophotometric measurements.

#### Fourier-transform infrared spectroscopy (FT-IR)

A Thermo Fisher Scientific Nicolet iS10 spectrometer (Ge/KBr beam splitter, DTGS detector) was used to conduct FT-IR measurements. 32 images were co-added at a spectral resolution of 4 cm^−1^ to gather data from 4000 to 1000 cm^−1^.

The pH levels were adjusted using a Hanna pH meter from Romania.

#### Molecular docking software

The Molecular Operating Environment (MOE) software, version 2022.20, was used to perform molecular docking simulations. Bovine Serum Albumin’s (BSA) three-dimensional crystal structure was obtained from the Protein Data Bank (PDB code: 4F5S). All computational work was performed in the Computational Chemistry and Molecular Modeling Lab at the Faculty of Pharmacy, Mansoura University^14,15^. For preparation, the BSA crystal structure was loaded into the Molecular Operating Environment (MOE) 2022.20 program. Hydrogen atoms were added after water molecules and heteroatoms were eliminated in this procedure. Concurrently, the ligand structure of cefpodoxime (CFP) was sketched in ChemDraw Ultra 17.1 and its three-dimensional geometry was generated and energetically minimized using the MOE’s molecular mechanics forcefield. The primary binding pocket on BSA was defined based on prior experimental evidence. The docking protocol was configured with the protein held in a rigid conformation. The “Triangle Matcher” algorithm was selected for generating ligand placement poses, which were then ranked using the “London dG” scoring function. A total of 30 independent docking runs were performed for CFP to ensure a comprehensive sampling of its binding orientation within the specified site.

### Procedures

#### Stock solutions preparation

All solutions were prepared daily in double-distilled water. Stock solutions of BSA (2 µM) and CFP (100 µM) were included, along with a 20 mM Tris-HCl buffer that had been pH-adjusted to 7.4 using 1.0 M HCl. To create working solutions, the stocks were diluted as necessary and kept at 4 °C.

#### UV-visible spectroscopic measurements

The investigation of the CFP-BSA interaction via UV-Vis absorption spectroscopy was conducted through a titration methodology. A constant, low concentration of BSA (4.0 × 10^−8^ M) was maintained throughout the experiment, while it was systematically titrated with incremental additions of a CFP stock solution, achieving a final concentration range of 0.4 × 10^−5^ M to 4.5 × 10^−5^ M. All samples were prepared in a 20 mM Tris-HCl buffer (pH 7.4) to maintain a consistent ionic strength and physiological pH, with the entire experiment conducted at a stabilized temperature of 298 K.

The absorption profiles for each resultant mixture were acquired across the ultraviolet and far-UV range of 190 to 350 nm to capture both peptide bond and aromatic amino acid absorptions. A critical step in data processing involved the correction of all collected spectra for background signal. This was accomplished by digitally subtracting from the raw spectra of the free BSA solution and each corresponding BSA-CFP complex the absorption spectrum of a matched Tris-HCl buffer blank^16^.

#### Quenching measurements

The quantitative assessment of the cefpodoxime (CFP) and bovine serum albumin (BSA) binding interaction was conducted through fluorescence titration. In this procedure, a solution containing a fixed concentration of BSA (4.0 × 10^−8^ M) was progressively titrated with microliter aliquots of a CFP stock solution, generating a set of samples with final CFP concentrations spanning from 0.4 × 10^−5^ M to 4.5 × 10^−5^ M. To enable the determination of thermodynamic parameters, the entire titration series was replicated in triplicate within a Tris-HCl buffer system (pH 7.4) at three distinct temperatures: 298 K, 310 K, and 318 K. For each sample, the intrinsic fluorescence of BSA was measured by recording the emission spectrum across a wavelength range of 280 to 500 nm after excitation at 285 nm capturing the quenching of BSA’s intrinsic fluorescence.

#### Synchronous fluorescence measurements

To monitor conformational changes in BSA upon binding, synchronous fluorescence spectroscopy was employed. Synchronous fluorescence (SF) spectra of BSA were recorded in the presence of increasing CFP concentrations (0 to 4.5 × 10^−5^ M). The spectra were scanned from 200 to 400 nm in Tris-HCl buffer (pH 7.4). To probe the local environment of specific fluorophores, wavelength intervals (Δλ) of 15 nm and 60 nm were used to characterize tyrosine and tryptophan residues, respectively.

#### Site markers competitive binding

Competitive binding tests were performed utilizing indomethacin and diazepam as particular site markers for Sudlow’s site I and II, respectively. In this procedure, a solution of BSA was pre-incubated with an equimolar concentration of a site marker for 30 min to ensure complex formation. After that, increasing concentrations of CFP were added to this pre-formed complex, and the resulting fluorescence emission spectra were measured.

#### FTIR method

Fourier-transform infrared (FTIR) spectra were acquired for free BSA (5 × 10⁻⁷ M) and its complex with CFP at a 1:1 molar ratio, focusing on the 1500–1800 cm^−1^ spectral region. The absorbance of free CFP and Tris buffer was recorded and digitally subtracted from the spectrum of the complex of BSA-CFP to separate the spectral contributions of the protein-ligand interaction and to ensure the observed signals originated solely from this interaction,

## Results and discussion

The degree of albumin binding has direct and profound implications for a drug’s therapeutic efficacy and distribution. Only the unbound, or free, fraction of a drug is pharmacologically active, capable of diffusing through capillary walls to reach its target tissues and exert a biological effect. To accurately characterize these critical interactions, we employed a suite of biophysical and computational techniques.

### UV-visible spectrophotometry

**Fig. 2:**
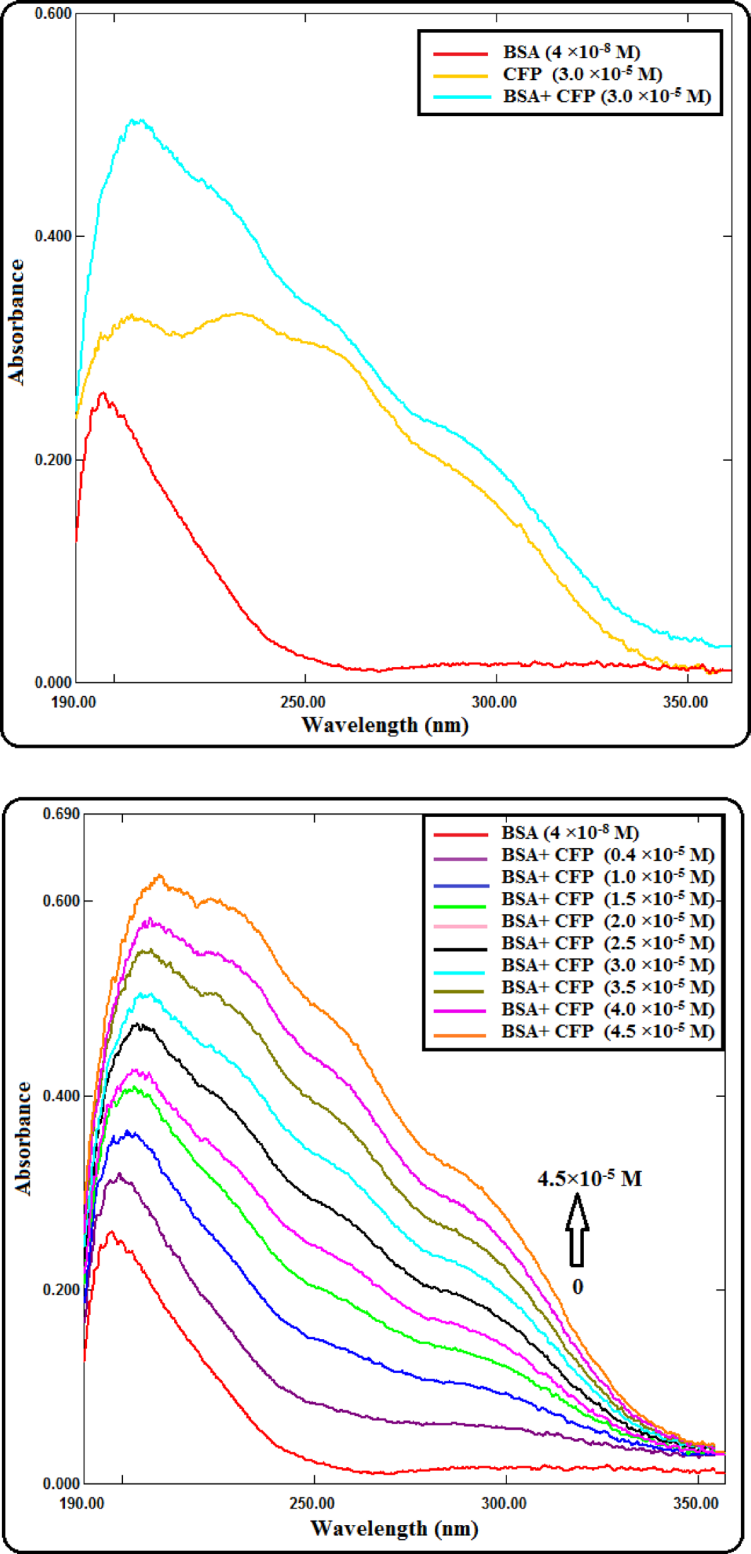
(**a**) Absorption spectra of BSA, CFP, and the CFP-BSA complex. Conditions: [BSA] = 4 × 10^−8^ M, [CFP] = 3.0 × 10^−5^ M, T = 298 K, pH 7.4. (**b**) Absorption spectral changes of BSA upon the addition of CFP. The spectrum of BSA (4 × 10^−8^ M) was recorded in Tris-HCl buffer (pH 7.4, 298 K) with CFP concentrations ranging from 0.4 to 4.5 × 10^−5^ M.

Alterations in protein conformation and drug-protein binding interactions can be effectively monitored using UV-visible absorption spectroscopy^17–19^. The absorption spectra of all samples were scanned between 190 and 350 nm. The background signal from the solvent was subtracted from the BSA and BSA-CFP complex spectra to eliminate its influence. The resulting protein spectra reflect the conformational state of BSA, as the microenvironment of its chromophores directly affects their absorption^20^. The aromatic amino acids tryptophan, tyrosine, and phenylalanine in BSA are responsible for the distinctive absorption peak at 278 nm, as seen in Fig. 2a. The intensity of this peak increases with increasing CFP concentrations (Fig. 2b), indicating the formation of a CFP-BSA complex. This hyperchromic shift indicates a reduction in the hydrophobicity around the chromophores, leading to a loosening and partial unfolding of the protein’s backbone structure. UV-Vis absorption spectra were recorded from 240 to 350 nm to monitor changes in the aromatic amino acid absorption. Due to the low concentration of BSA used ($\:4\times\:{10}^{-8}{\hspace{0.17em}M}$), measurements in the far-UV region (below 230 nm) were not feasible for reliable secondary structure analysis. To confirm that spectral changes arise from binding rather than additive absorbance, the spectrum of free CFP was subtracted from each BSA–CFP mixture after buffer blank correction. The resulting differential spectra (Supplementary Fig. S1) show clear hyperchromicity at 278 nm without isosbestic points, confirming ground-state complex formation rather than mere spectral overlap.

### Spectrofluorimetric method

**Fig. 3:**
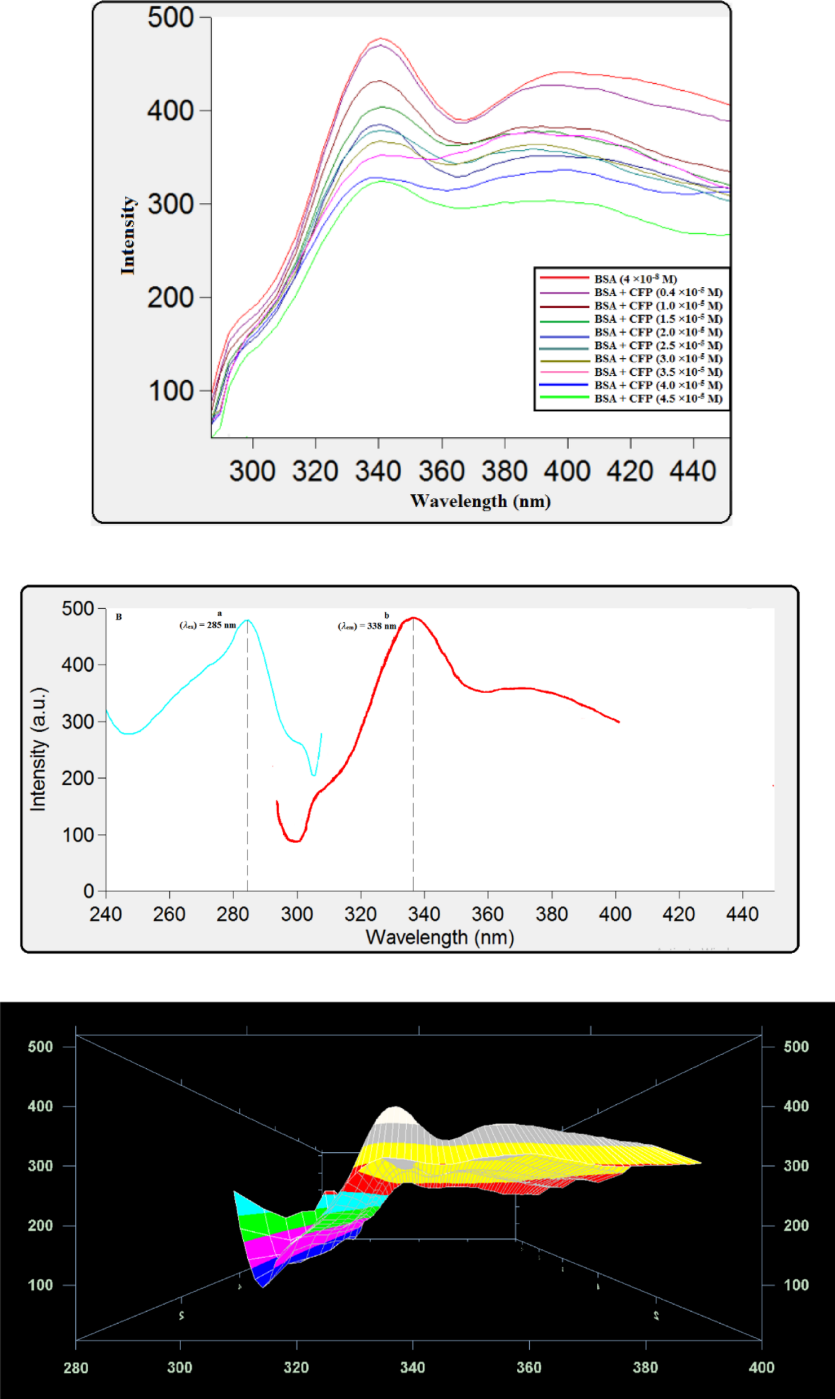
(**A**) Fluorescence quenching of BSA (4 × 10^−8^ M) by CFP (0.4–4.5 × 10^−5^ M) at pH 7.4, 298 K. (**B**) Excitation (a) and emission (b) spectra for BSA (4 × 10^− 8^ M) at *T* = 298 K and *pH* = 7.4, and (*λ*_ex_) = 285 nm. (**C**) 3D fluorescence spectral changes of BSA induced by CFP binding. Spectra were recorded for BSA (4 × 10^−8^ M) titrated with CFP (0.4–4.5 × 10^−5^ M) in Tris-HCl buffer (pH 7.4, 298 K).

Using this method, the CFP-BSA binding process was investigated, and the thermodynamic parameters, site multiplicity, and binding constant were measured. The interaction was monitored by measuring the reduction in the protein’s intrinsic fluorescence intensity at its emission maximum^12,21^. As visually demonstrated in Fig. 3A and B, and 3C, a systematic, concentration-dependent reduction in BSA fluorescence was observed with each successive addition of CFP. The emission peak above 360 nm in the original spectra was attributable to Raman scattering from the Tris-HCl buffer. The fluorescence signal originates predominantly from the aromatic side chains of specific amino acids within the protein’s structure; BSA contains two tryptophan and eighteen tyrosine residues, which are the primary fluorophores excited at the wavelength of 285 nm^22^.

When increasing concentrations of cefpodoxime (CFP) were added, the intrinsic fluorescence emission of bovine serum albumin (BSA) showed a progressive and systematic drop in intensity, indicating a clear concentration-dependent quenching relationship. To quantitatively analyze this phenomenon and derive the binding affinity, the data obtained at each experimental temperature were fitted to the Stern-Volmer model. This established analytical framework was utilized to calculate the respective Stern-Volmer quenching constants (K_SV), thereby providing a quantitative measure of the interaction strength across the different thermal conditions.

1 $$\:\frac{F0\:}{F}=1+{K}_{sv}\left[Q\right]$$

F_o_ and F: fluorescence intensities of BSA before and after binding with CFP.

K_SV_ is the Stern-Volmer constant and.

[Q] is the concentration of the quencher.

The fluorescence quenching showed a clear dependence on the CFP concentration. The Stern-Volmer equation was used to quantify this relationship and ascertain the binding parameters at the different temperatures under investigation^12,23^. The temperature dependence of the quenching constant can distinguish the mechanism: an increase suggests dynamic quenching (faster diffusion), while a decrease indicates static quenching (reduced complex stability)^24^. The magnitude of the quenching rate constant (kq) indicates a static quenching mechanism, which involves the formation of a complex. The constant was computed using the following equation:

2 $$\:\mathrm{K}\mathrm{q}=\frac{\mathrm{K}\mathrm{S}\mathrm{V}}{{{\uptau\:}}_{0}}$$

In this equation, kq is the bimolecular quenching rate constant and τ_0_ is the average excited-state lifetime of the fluorophore. For this biomacromolecule (BSA), a value of τ_0_ = 10^−8^ s was employed^12,25,26^.

**Fig. 4:**
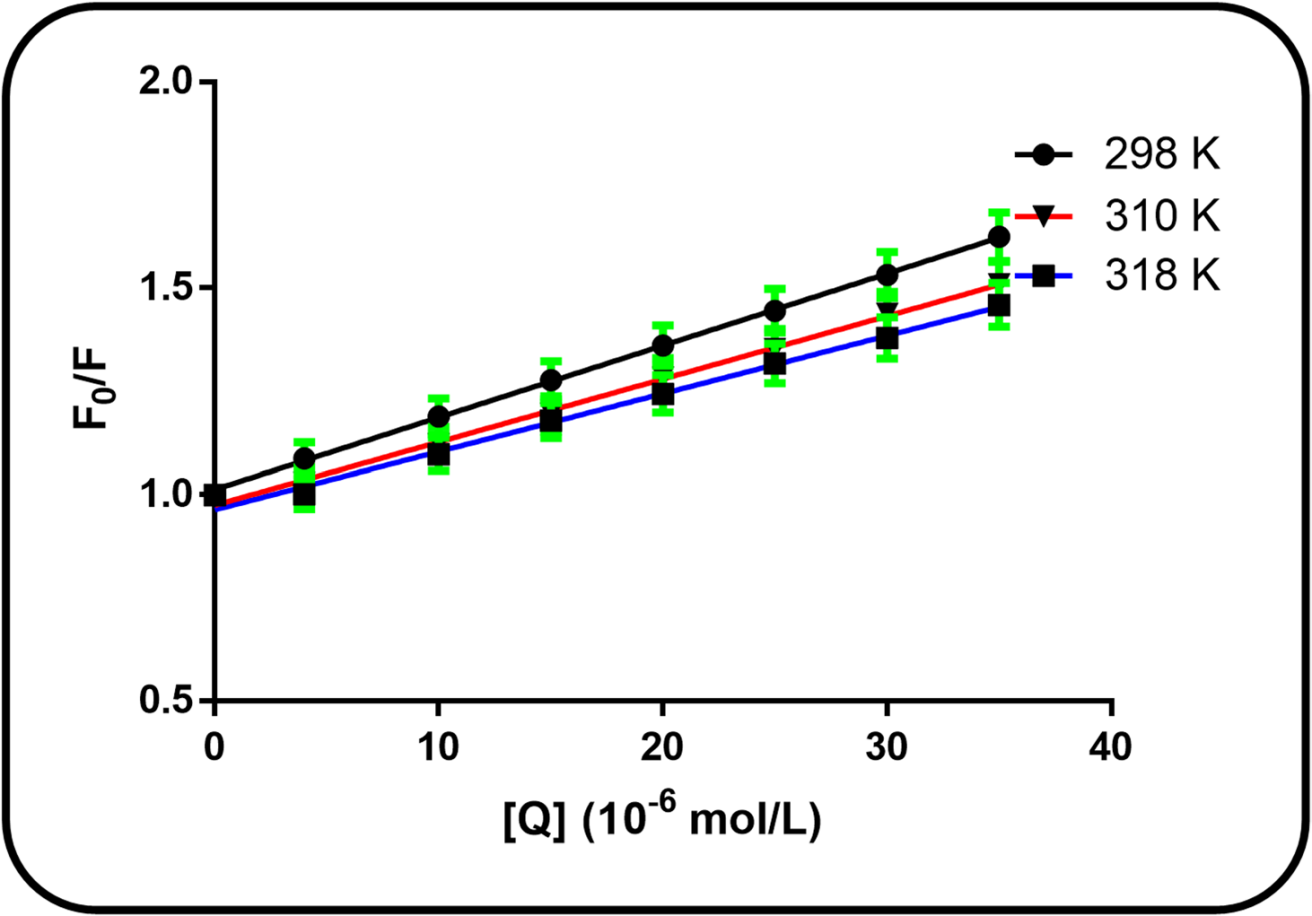
Stern-Volmer plots illustrating the concentration-dependent fluorescence quenching of BSA (4 × 10^−8^ M) by CFP at three different temperatures (298, 310, and 318 K).

The Stern-Volmer plots for the fluorescence quenching of BSA by CFP at different temperatures are presented in Fig. 4, with the corresponding KSV and kq values listed in Table 1. The calculated bimolecular quenching rate constant (kq) was on the order of 10^12^ L mol^−1^ s^−1^, which substantially exceeds the maximum value for diffusion-controlled quenching (2 × 10^10^ L mol^−1^ s^−1^). This confirms that the quenching mechanism is static, indicating the formation of a ground-state complex^27^, suggesting occurrence of static quenching of the interaction between CFP and BSA^28,29^.

**Table 1:** Stern-Volmer quenching constants (K_SV) and bimolecular quenching rate constants (k_q) for the CFP-BSA interaction at 298, 310, and 318 K:

T (K)	K_sv_ × 10^4^ (Lmol^− 1^)	k_q_ × 10^12^ (Lmol^−1^s^− 1^)	*r* ^2^	SD	%Error
298	2.124	2.124	0.9969	0.723	0.323
310	1.527	1.527	0.9994	0.512	0.229
318	1.276	1.276	0.9974	0.580	0.259

For static quenching, $\:{K}_{SV}$ approximates the binding constant. The close agreement between $\:{K}_{SV}$ (2.12 × 10^4^ M^−1^) and $\:{K}_{b}$ (3.99 × 10^4^ M^−1^) at 298 K further supports a static quenching mechanism, as both constants reflect the same ground-state association.

To estimate the fraction of accessible fluorophores, the quenching data were analyzed using the modified Stern-Volmer equation:

3 $$\:\frac{{\mathrm{F}}_{0}}{{\mathrm{F}}_{0}-\mathrm{F}}=\frac{1}{{\mathrm{f}}_{\mathrm{a}}{\mathrm{K}}_{\mathrm{S}\mathrm{V}}\left[\mathrm{Q}\right]}+\frac{1}{{\mathrm{f}}_{\mathrm{a}}}$$

where $\:{\mathrm{f}}_{\mathrm{a}}$ is the accessible fraction. The calculated $\:{\mathrm{f}}_{\mathrm{a}}$ values were about 0.85–0.90 across temperatures, indicating that most fluorophores are accessible to CFP, consistent with binding in the hydrophobic pocket of Site I^44^.

It is important to note that the distinction between static and dynamic quenching is most definitively made using time-resolved fluorescence lifetime measurements. In a pure static mechanism, the fluorescence lifetime of the protein remains unchanged with ligand binding, while dynamic quenching leads to a measurable reduction in lifetime. Our conclusion of static quenching is based on strong evidence from steady-state measurements, the inverse temperature dependence of $\:{K}_{SV}$ and the magnitude of $\:{k}_{q}$ exceeding the diffusion-controlled limit. Future studies employing lifetime measurements could provide clear confirmation of this mechanism.

### Estimation of binding constant and stoichiometry

For static quenching interactions, the binding constant (K_a_) is calculated to quantify the affinity between the ligand and the protein, based on the correlation between the fluorescence intensity and the quencher concentration^30^. The following double-logarithmic equation was used to calculate the binding constant and the number of binding sites:

4 $$\:\mathrm{log}\frac{{F}_{o}-F}{F}=\mathrm{log}{K}_{b}+n\:log\:\left[Q\right]\:\:$$

**Fig. 5:**
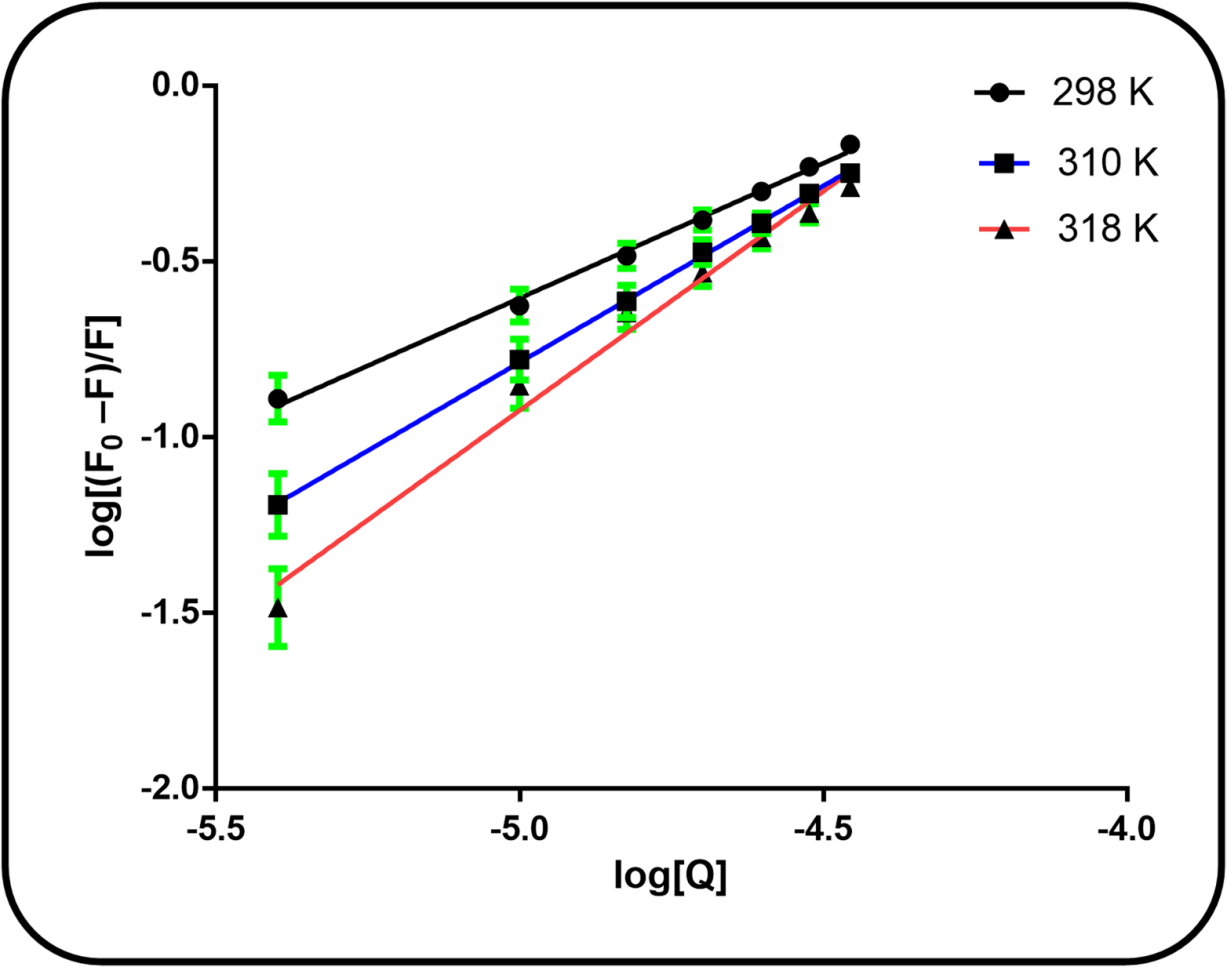
Double logarithmic plots for the CFP-BSA complex system at 298 K, 310 K and 318 K.

To quantify the binding affinity and stoichiometry of the cefpodoxime-BSA complex, the fluorescence quenching data were further analyzed using the double-logarithmic model. The binding constant (K_b) and the number of binding sites (n) were determined from the slope and the y-intercept, respectively, of the linear plot of log[(F_0_ - F)/F] against log[Q], as presented in Fig. 5. The results, compiled in Table 2, demonstrate that the value of n remains close to unity at each of the investigated temperatures (298, 310, and 318 K). This near-integer value signifies that a single primary binding site on BSA is occupied by CFP. Furthermore, the calculated binding constants were consistently found to be within the 10^4^ M^−1^ range, which is indicative of a moderately strong binding affinity between the drug and the serum albumin protein.

**Table 2:** Binding parameters for the CFP-BSA interaction at different temperatures.

T (K)	K_b_ × 10^4^ (Lmol^−1^)	*n*	*r* ^2^	SD	%Error
298	3.99	0.808	0.9959	0.072	0.032
310	1.923	1.057	0.9995	0.138	0.062
318	1.282	1.312	0.9844	0.233	0.104

### Thermodynamics characteristics and binding forces

The formation of drug-protein complexes is typically stabilized by four types of intermolecular forces: hydrophobic interactions, hydrogen bonding, van der Waals forces, and electrostatic interactions^27^. The thermodynamic parameters, specifically the enthalpy change (ΔH°) and entropy change (ΔS°), can be used to infer the type of binding forces present in the reaction. The correlation between these parameters and the primary interaction forces was established by Ross and Subramanian^31^. The predominant binding forces can be identified based on the sign and magnitude of the thermodynamic parameters. According to the framework established by Ross and Subramanian, negative values for both ΔH° and ΔS° suggest that van der Waals interactions and hydrogen bonding are dominant. In contrast, positive values for both ΔH° and ΔS° are characteristic of hydrophobic interactions. Electrostatic forces are typically indicated when ΔH° is negative and ΔS° is positive. For systems with minimal heat capacity change, the Van’t Hoff equation can be applied to determine the values of ΔH° and ΔS°^31,32^.

5 $$\:\mathrm{ln}{K}_{b}=-\frac{{\:\varDelta\:\mathrm{H}}^{\mathrm{o}}}{\mathrm{R}\mathrm{T}}+\frac{{\varDelta\:\mathrm{S}}^{\mathrm{o}}}{\mathrm{R}}$$

The binding constant (Kb) was determined at the corresponding absolute temperature (T), and R is the universal gas constant.

**Fig. 6:**
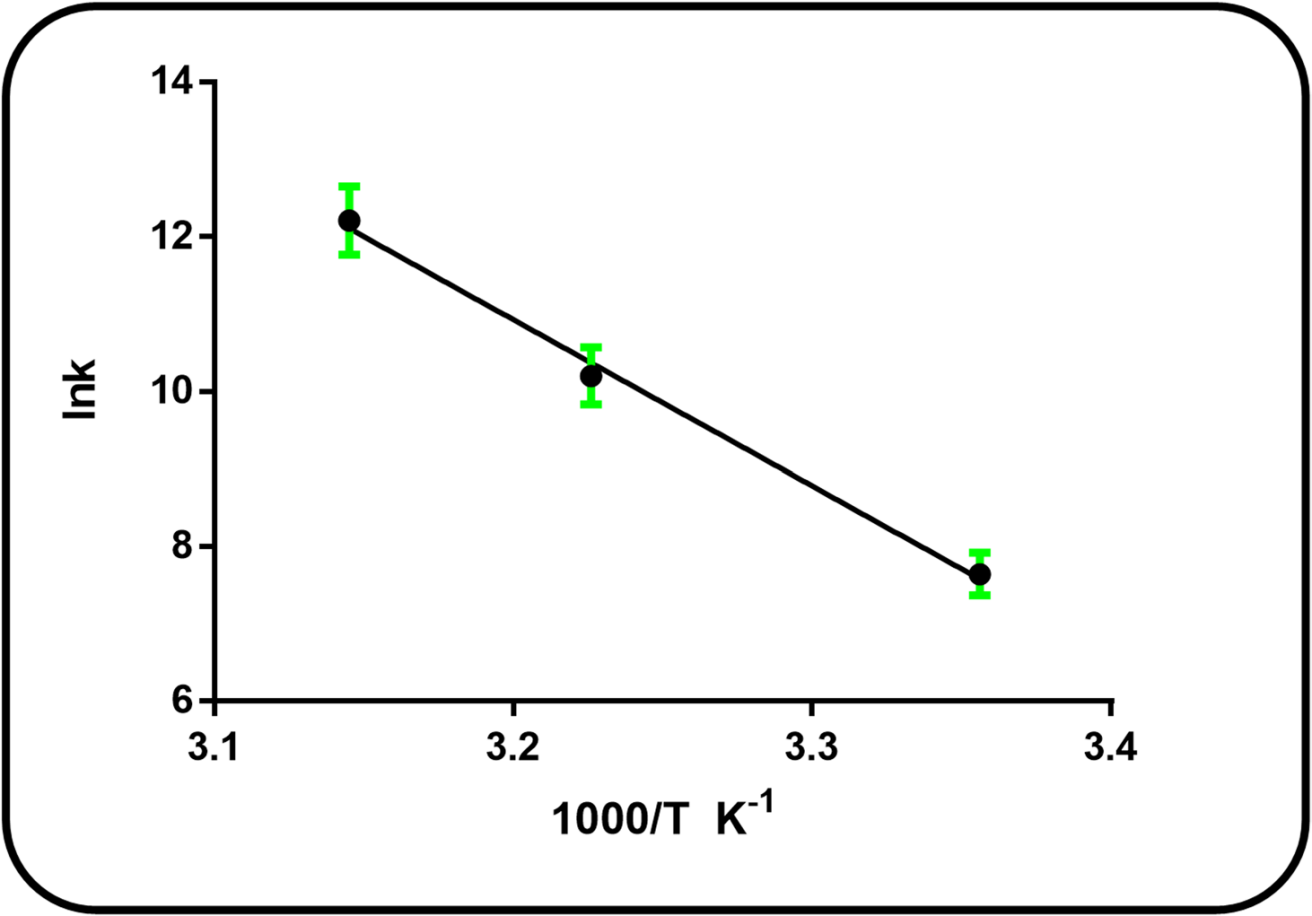
Van’t Hoff plot for binding of CFP with BSA.

The Van’t Hoff plot of lnKb versus 1/T yielded a linear relationship (Fig. 6), from which the standard enthalpy change (ΔH°) and standard entropy change (ΔS°) were calculated from the slope and the y-intercept, respectively. The standard Gibbs free energy change (ΔG°) was then computed using the following equation:

6 $$\:{\varDelta\:G}^{\mathrm{o}}=\:{\varDelta\:H}^{\mathrm{o}}-T$$

According to Table 3, the negative value of ΔG° verifies that CFP binding to BSA occurs spontaneously. The interaction is endothermic and mostly driven by hydrophobic forces, as indicated by the positive values for both ΔH° and ΔS°.

**Table 3:** Thermodynamic parameters of CFP-BSA interaction at pH 7.4:

T (K)	ΔH° (kJ mol^−1^)	ΔG° (kJ mol^−1^)	ΔS° (J mol^−1^K^−1^)	*r* ^2^
298	182.899	-19.27	678.42	0.9958
310		-27.41		
318		-32.84		

### Synchronous fluorescence spectroscopy

**Fig. 7:**
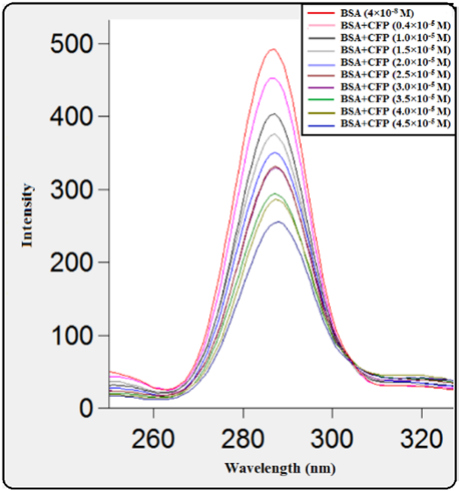
Synchronous fluorescence spectra of BSA (4 × 10^− 8^ M) with different concentrations of CFP (×10^− 5^ M): (0.4, 1, 1.5, 2, 2.5, 3, 3.5, 4, 4.5) at Δλ of 15 nm.

**Fig. 8:**
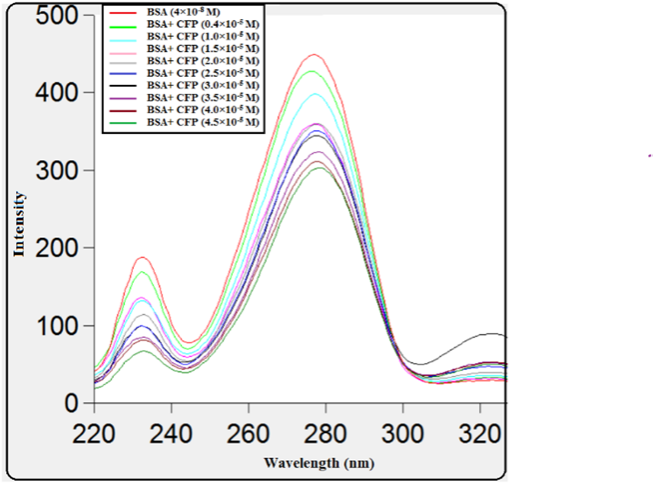
Synchronous fluorescence spectra reflecting the microenvironment of tryptophan residues (Δλ = 60 nm) in BSA (4 × 10^−8^ M) upon titration with CFP (0.4–4.5 × 10^−5^ M).

The application of synchronous fluorescence spectroscopy allows for the analysis of protein microenvironments by monitoring shifts in emission wavelength^33^. Synchronous fluorescence spectra were recorded at Δλ = 15 nm and 60 nm to selectively probe the microenvironments of tyrosine and tryptophan residues in BSA, respectively. As shown in Fig. 7, the emission maximum for tyrosine (Δλ = 15 nm) remained largely unchanged upon CFP binding. In contrast, the tryptophan spectrum (Δλ = 60 nm, Fig. 8) exhibited a slight blue shift from 277 nm to 275 nm. This shift indicates a decrease in the polarity surrounding the tryptophan residues, suggesting their insertion into a more hydrophobic environment upon complex formation with CFP. The multiple peaks in the synchronous fluorescence spectra at Δλ = 60 nm arise from the vibrational fine structure of tryptophan residues in BSA, a known feature in synchronous scanning mode. The observed blue shift in the dominant peak (277 → 275 nm) indicates reduced polarity around tryptophan upon CFP binding. These spectral changes collectively confirm that the binding of CFP induces a conformational change in BSA^34^.

### Site markers competitive binding

**Fig. 9:**
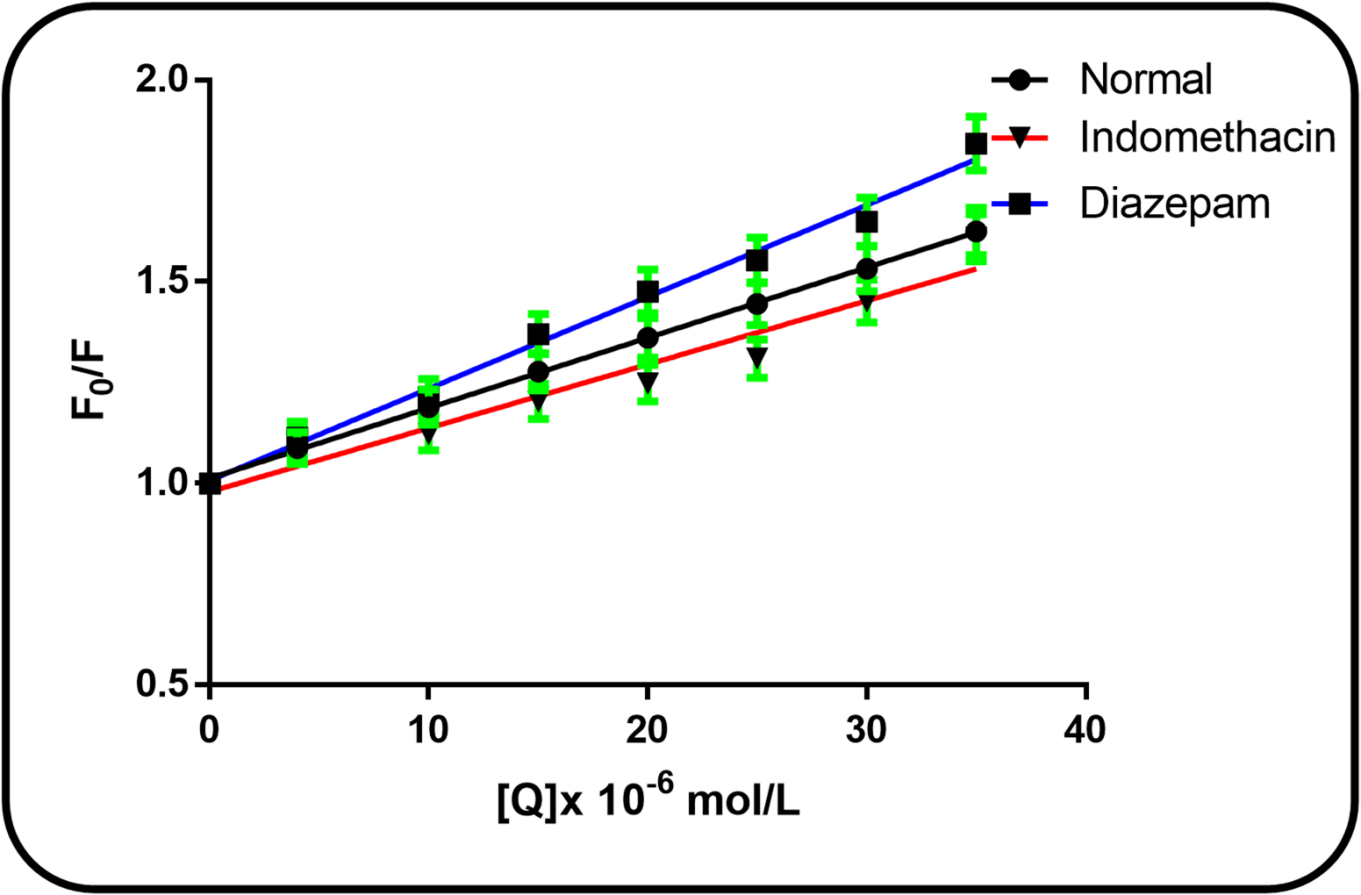
Stern-Volmer plots for the fluorescence quenching of BSA by CFP alone and in the presence of site markers indomethacin (Site I) and diazepam (Site II).

Each of the three homologous domains (I, II, and III) that make up the structure of BSA is made up of two subdomains (A and B)^3,35^. Competitive binding experiments with site-specific markers were conducted to identify the primary binding site of CFP on BSA. Based on the classical definition by Sudlow et al.^36^, the major drug-binding sites on albumin are Site I (located in subdomain IIA) and Site II (located in subdomain IIIA), which are specifically bound by warfarin/indomethacin and diazepam, respectively. In the presence of each marker, the CFP-BSA complex’s binding constant was determined (Table 4). When indomethacin (Site I marker) was present, the binding constant significantly decreased, whereas diazepam (Site II marker) had no effect. This indicates that CFP binds specifically to Sudlow’s Site I in subdomain IIA. This conclusion is consistent with the molecular docking results presented in Sect. 3.9^37,38^. When indomethacin (Site I marker) was present, the Stern-Volmer constant ($\:{K}_{SV}$) decreased significantly, confirming competition for Site I. In contrast, the presence of diazepam (Site II marker) resulted in a modest increase in $\:{K}_{SV}$, suggesting a potential allosteric interaction or displacement from a secondary binding site (Fig. 9).

**Table 4:** Stern-Volmer constants (K_sv_) and bimolecular quenching rate constants (k_q_) for the CFP-BSA interaction in the absence and presence of site markers:

Site marker	K_sv_ (10^4^ L mol^− 1^)	k_q_ (10^12^ L mol^− 1^ s^− 1^)	SD	%Error
BSA + CFP	2.124	2.124	0.723	0.323
BSA + CFP+IND	1.773	1.773	1.004	0.449
BSA + CFP+DIA	2.641	2.641	0.706	0.316

### FTIR spectroscopy

**Fig. 10:**
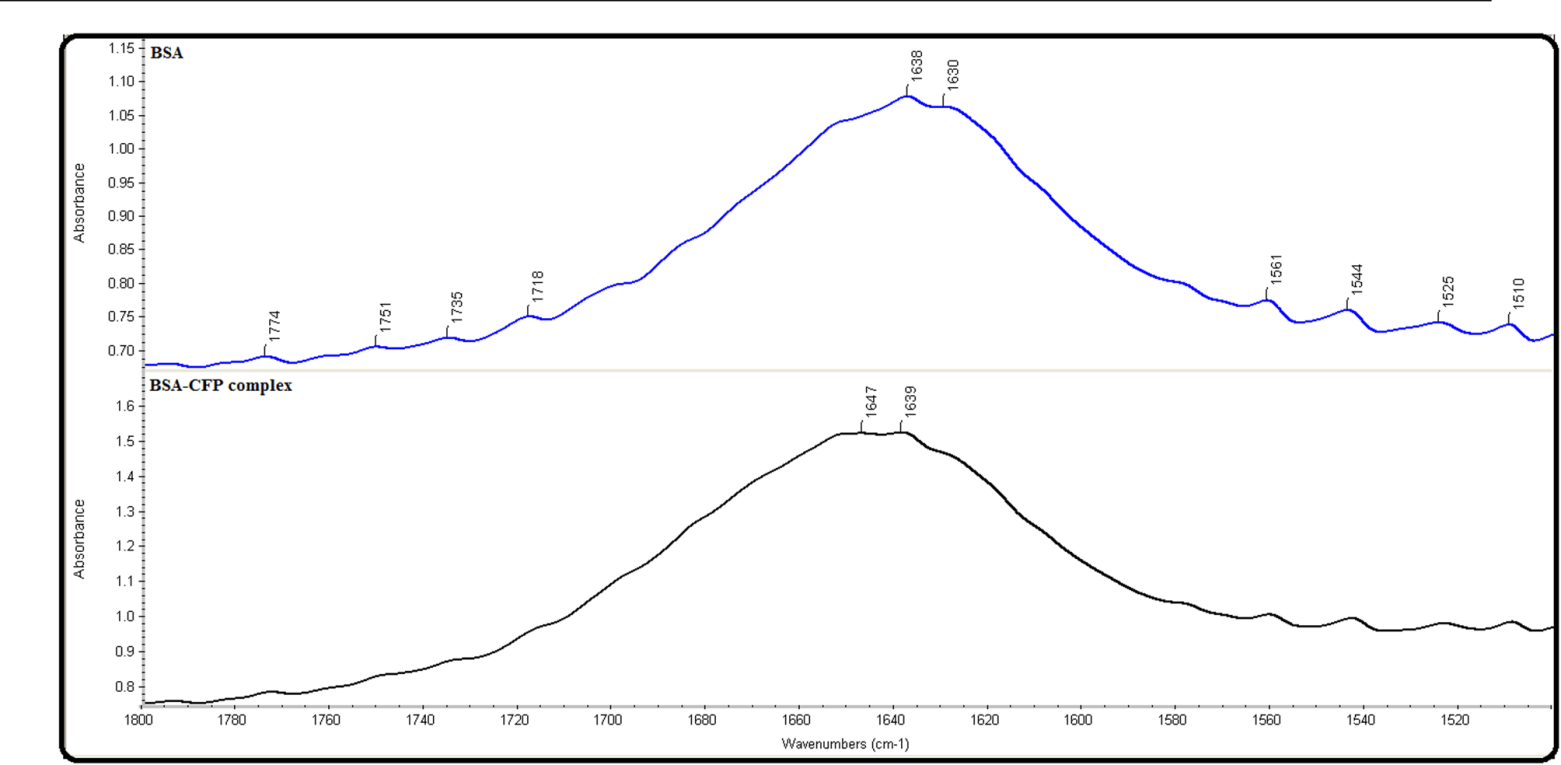
FTIR Spectra of BSA and CFP-BSA complex at 298 K and *pH* of 7.4.

Fourier-Transform Infrared (FTIR) spectroscopy is an effective method for examining the dynamics of proteins and describing their secondary structure^39^. The FTIR spectra in Fig. 10 confirm the interaction between CFP and BSA. The protein’s infrared spectrum is dominated by amide I and amide II bands, arising from the vibrations of the peptide backbone. The amide I band (1600–1700 cm^−1^) is highly sensitive to secondary structure and is more diagnostically useful than the amide II band (1500–1600 cm^−1^). Upon complex formation with CFP, the amide I peak position shifted, indicating a change in BSA’s secondary structure. Specifically, the peak shifted from 1630 cm^−1^ to 1639 cm^−1^, and from 1638 cm^−1^ to 1647 cm^−1^, confirming a conformational alteration in the protein^40^.

### Fluorescence resonance energy transfer (FRET)

**Fig. 11:**
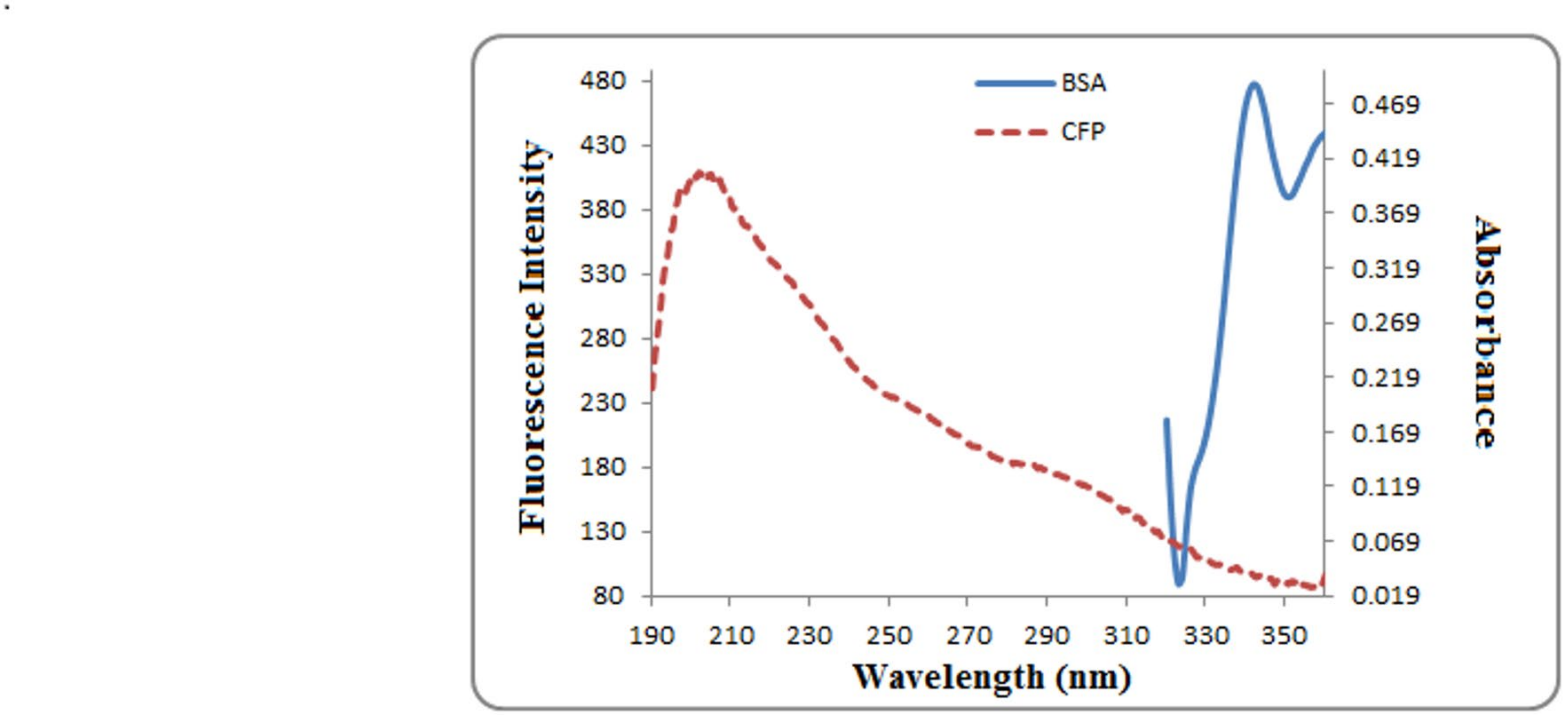
Spectral overlap between the fluorescence emission spectrum of BSA (4 × 10^−8^ M) and the UV-Vis absorption spectrum of CFP (1.5 × 10^−5^ M), which is essential for Förster Resonance Energy Transfer (FRET).

The Förster Resonance Energy Transfer (FRET) mechanism involves the non-radiative transfer of energy from a donor molecule (BSA) to an acceptor molecule (CFP) over a distance. This resonance occurs through dipole-dipole coupling between the fluorophores and is highly dependent on their separation, requiring no molecular collision or conversion to thermal energy. According to Förster’s theory, the efficiency of this energy transfer is governed by several key factors^41^:

The efficiency of Förster Resonance Energy Transfer (FRET) is governed by several key parameters, as per the established theory:

1. A notable overlap between the acceptor’s UV absorption spectrum and the donor’s fluorescence emission spectrum (Fig. 11).
2. A large spectral overlap integral (J), which quantifies the extent of this overlap.
3. Favorable relative orientation between the transition dipoles of the donor and acceptor molecules.

The energy transfer efficiency (E) was calculated using the following equation:

7 $$\:E=1-\frac{F}{{F}_{o}}=\frac{{R}_{o}^{6}}{{R}_{0}^{6}{+\:r}^{6}}$$

The energy transfer efficiency (E) was determined from the fluorescence intensities of BSA before (F_0_) and after (F) the addition of CFP, according to Eq. (7). The calculation relates the actual donor-acceptor distance (R) to the Förster distance (R_0_), which is defined as the separation where the energy transfer efficiency is 50%.

The value of R_0_ was determined using Eq. (8):

8 $$\:{R}_{o}^{6}=8.8\times\:{10}^{-25}{k}^{2}{N}^{-4}\varPhi\:J$$

where κ^2^ is the orientation factor (assumed to be 2/3 for dynamic random averaging), N is the refractive index of the medium (1.336), Φ is the fluorescence quantum yield of BSA (0.15), and J is the spectral overlap integral.

The overlap integral (J) was calculated from Eq. 9 by integrating over the wavelength range of 300–450 nm:

9 $$\:J=\frac{\int\:F\left(\lambda\:\right)\epsilon\:\left(\lambda\:\right){\lambda\:}^{4}\varDelta\:\lambda\:\:}{\int\:F\left(\lambda\:\right)\varDelta\:\lambda\:}$$

where F(λ) is the fluorescence intensity of BSA (donor) at wavelength λ, and ε(λ) is the molar absorptivity of CFP (acceptor) at wavelength λ. The computed value for J was 5.72 × 10^−14^ cm^3^ L mol^−1^^42^. , *E* = 0.3, *R*_*o*_ = 3.41 nm, and *r* = 3.93 nm can be calculated from Eq. (7)–(9). The average distance between BSA and CFP is less than 8 nm proposing the occurrence of energy transfer^43^.

### Molecular docking

**Fig. 12:**
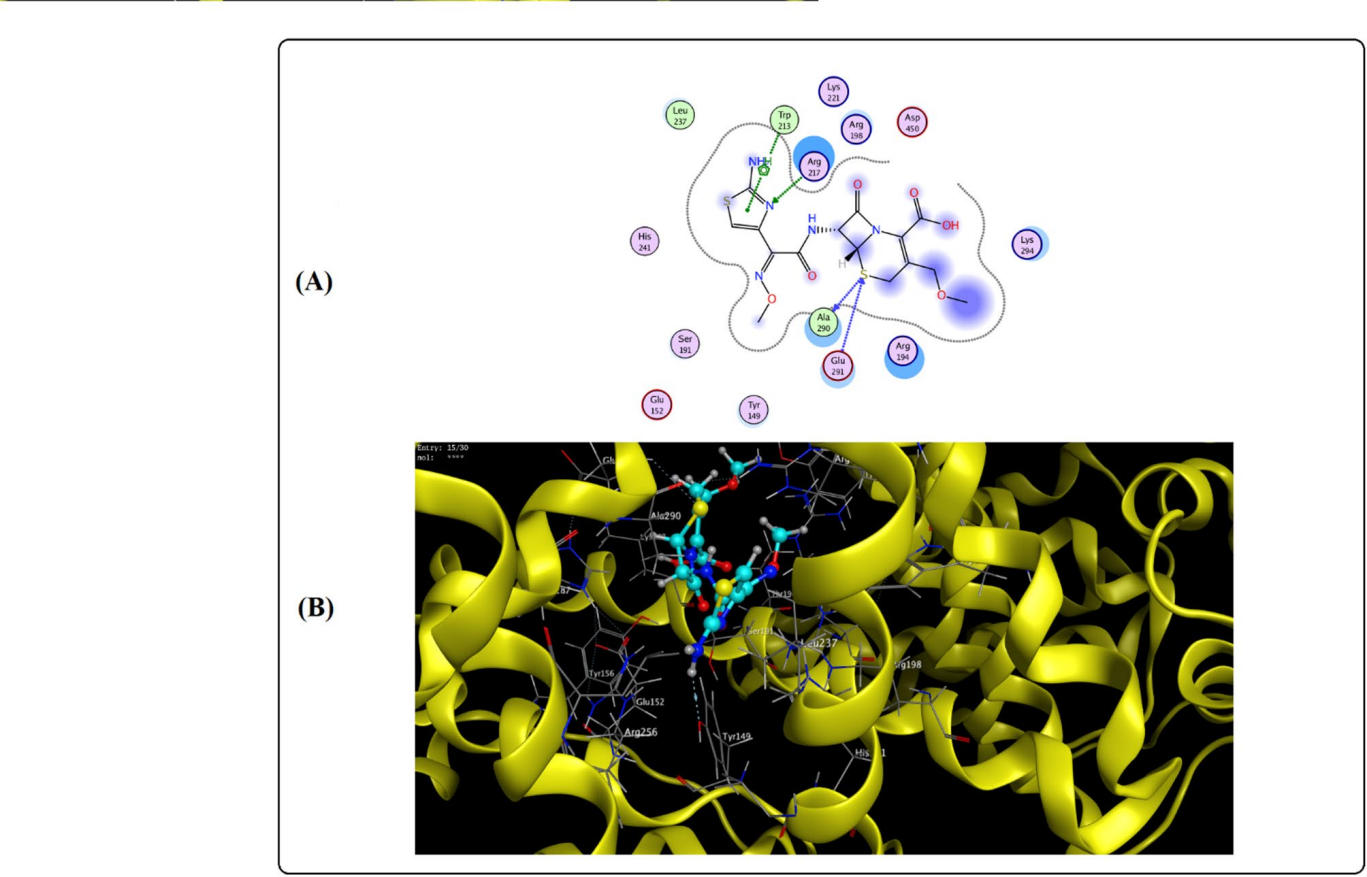
(**A**) Diagram showing the amino acid residues included in the binding pocket of BSA site 1 that are involved in the CFP-BSA interaction. (**B**) Three-dimensional structure of the amino acid residues included in the CFP-BSA interaction inside the BSA site 1 binding pocket.

Molecular docking simulations were employed to validate the experimental findings regarding CFP’s binding affinity and interaction mode with BSA. The results corroborate the competitive site-marker studies, confirming that CFP binds specifically to Site I within subdomain IIA (Fig. 12A and B). The docking pose with the highest affinity yielded a score of -8.5 kcal/mol (Fig. 12A). Analysis of the binding profile revealed that the interaction is stabilized by several key forces. A crucial arene-arene interaction was observed between the thiazole ring of CFP and the Trp213 residue. Additional hydrophobic contacts involved Ala290 and Glu291 with the 1,3-thiazine ring. Furthermore, binding was reinforced by hydrogen bonds with Arg217 and Tyr149. These computational results are in excellent agreement with the experimental thermodynamic data, confirming that hydrophobic interactions are the primary driving force for the CFP-BSA complex formation.

The docking score (− 8.5 kcal/mol) correlates well with the experimental ΔG° (− 19.27 kJ/mol), confirming favorable binding. The predicted hydrophobic interactions with Trp213, Ala290, and Glu291 align with the positive ΔS° observed. Hydrogen bonds with Arg217 and Tyr149 further stabilize the complex. These computational insights reinforce the experimental conclusion that hydrophobic forces dominate, and CFP binds specifically to Site I.

Finally, Compared to other cephalosporins such as cefotaxime and ceftriaxone, which show higher binding constants (~ 10^5^ M^−1^), cefpodoxime exhibits moderate affinity, consistent with its lower plasma protein binding. This places it within the range of rapidly equilibrating antibiotics, supporting its pharmacokinetic profile.

## Conclusion

This research presents a comprehensive mechanistic analysis of the molecular interaction between the antibiotic cefpodoxime (CFP) and bovine serum albumin (BSA), employing an integrated strategy of in-vitro spectroscopy and in-silico simulation under physiologically relevant conditions. The convergence of data from multiple analytical techniques consistently demonstrated that CFP forms a stable, spontaneously formed 1:1 complex with BSA through a static quenching process. Thermodynamic profiling of the interaction, characterized by a negative free energy change (ΔG°) and a positive entropy change (ΔS°), established that the binding is energetically favorable and predominantly mediated by hydrophobic effects. The precise binding locus was unequivocally mapped to the hydrophobic pocket of Sudlow’s Site I in subdomain IIA, a finding robustly confirmed by both competitive ligand displacement assays and computational docking.

The moderate binding affinity (Kb ~ 10^4^ M^−1^) and spontaneous complex formation observed in this study align with the reported low plasma protein binding (18–23%) of CFP. This suggests that while CFP interacts with serum albumin, a significant fraction remains unbound, consistent with its extensive tissue distribution and renal clearance. The identification of Site I (subdomain IIA) as the binding locus further supports its transport mechanism in plasma, potentially affecting its free concentration and availability for antibacterial action. These insights help rationalize its pharmacokinetic behavior, including its half-life (~ 2 h) and susceptibility to changes in renal function.

Collectively, these results offer pivotal insights into the pharmacokinetic behavior of CFP, specifically detailing its transport modality within the circulatory system. By delineating the fundamental nature of this drug-protein complex, this study provides a crucial experimental foundation for rationally evaluating key pharmacological attributes, including bioavailability and potential toxicity, thereby informing strategies for its safer and more effective clinical deployment. Furthermore, the integrated methodological approach validated in this work establishes a robust template for probing the plasma protein binding dynamics of other cephalosporin antibiotics or drug candidates. The strong correlation observed between experimental and computational data underscores the power of combining these techniques to rapidly and accurately characterize supramolecular interactions. Future investigations could leverage this framework to conduct comparative studies across different antibiotic classes, which would be instrumental in constructing predictive models of drug disposition.

## Supplementary Information

Below is the link to the electronic supplementary material.

Supplementary Material 1

## Data Availability

Data will be made available upon request from the corresponding author.
