# Supplementary material for: Elucidating the mechanism of cefpodoxime-BSA interaction via a combination of multi-spectroscopic methods and molecular docking simulations
Source: Sci Rep. 2026 Mar 1;16:7836. doi: 10.1038/s41598-026-39137-8 (PMC12953881; doi:10.1038/s41598-026-39137-8)
Supplement: Supplementary file 1 — Supplementary Material 1 [file 41598_2026_39137_MOESM1_ESM.docx]

**Figure S1** showing the subtracted UV spectra of free CFP from each BSA–CFP mixture after buffer blank correction

**Figure S2 : Spectra of :**

- BSA + site marker
- BSA + site marker + CFP titration
- Overlay of quenching profiles


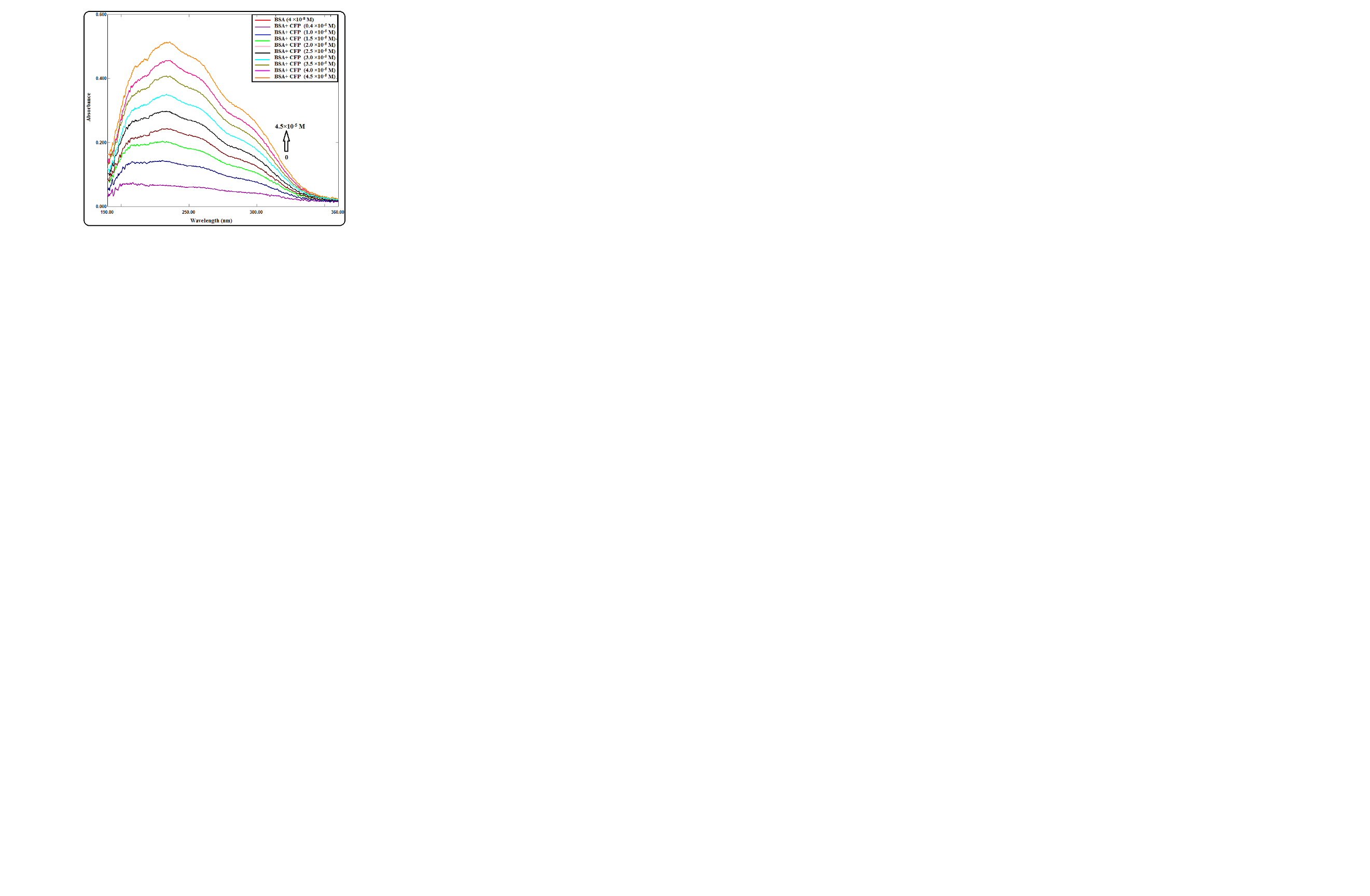


Figure S1


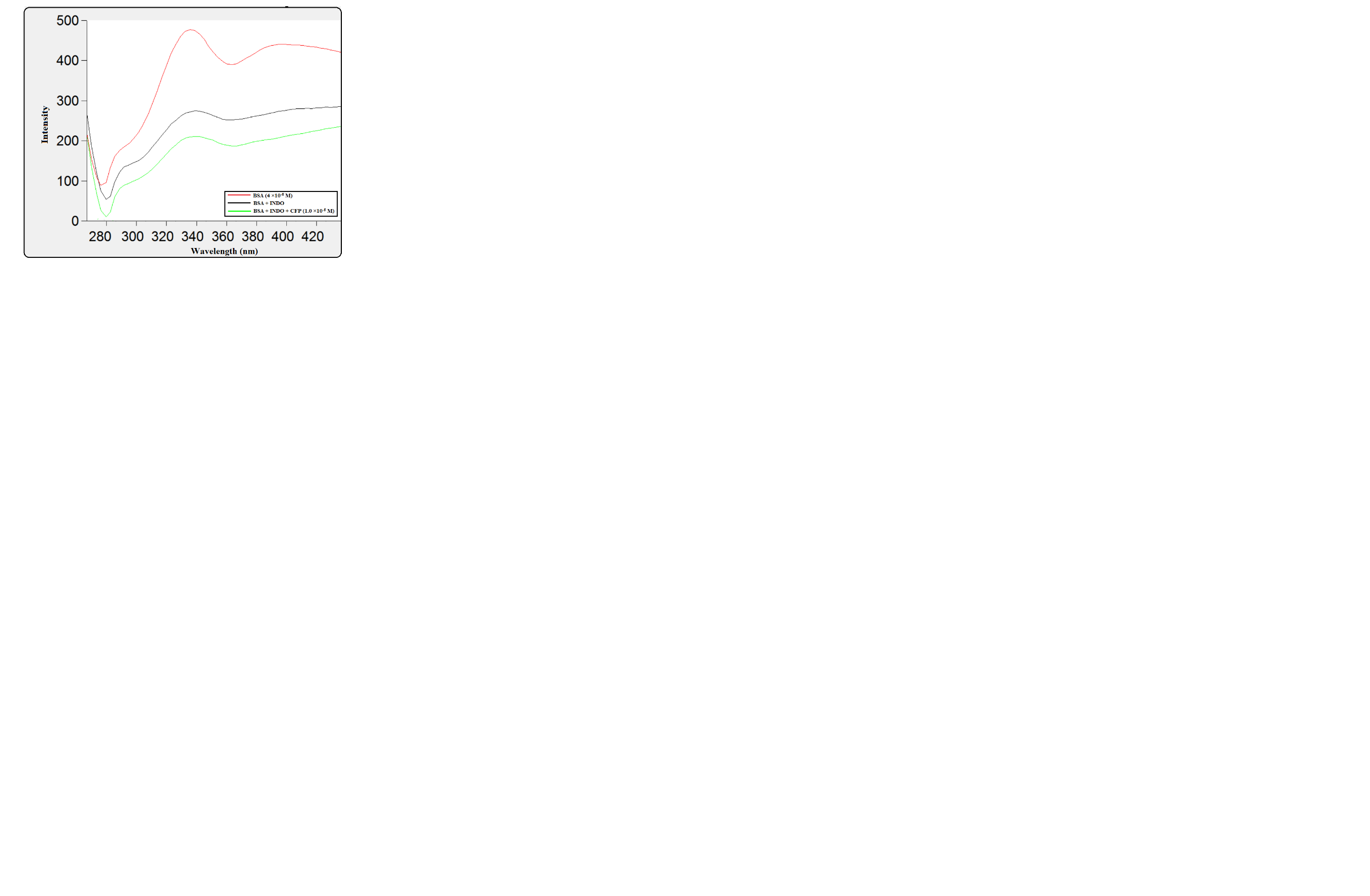


Figure S2
